# Invisible Ink for Postals

**Published:** 1878-06

**Authors:** 


Invisible Ink for Postals.—John H. Nelson gives in his "Hand-Book of Formula" the following:

Oxide of cobalt, . . . . . ½ ounce.
Muriatic acid, sufficient to dissolve it.
Water, . . . . . . . 4 ounces.
Mucilage of gum acacia, . . . 1 drachm.

Characters written on paper with this solution are invisible, but on the application of heat they instantly appear in blue; on cooling they become invisible again. —*Phil. Druggist and Chemist.*

